# RGD-conjugated silica-coated gold nanorods on the surface of carbon nanotubes for targeted photoacoustic imaging of gastric cancer

**DOI:** 10.1186/1556-276X-9-264

**Published:** 2014-05-27

**Authors:** Can Wang, Chenchen Bao, Shujing Liang, Hualin Fu, Kan Wang, Min Deng, Qiande Liao, Daxiang Cui

**Affiliations:** 1 Xiangya Hospital of Central South University, 87 Xiangya Road, Changsha Hunan 410008, People's Republic of China; 2 Institute of Nano Biomedicine and Engineering, Key Laboratory for Thin Film and Microfabrication Technology of the Ministry of Education, Department of Instrument Science and Engineering, Research Institute of Translation Medicine, Shanghai JiaoTong University, Dongchuan Road 800, Shanghai 200240, People's Republic of China

**Keywords:** RGD peptide, Gold nanorods, Multiwalled carbon nanotubes, Optoacoustic imaging, Gastric cancer, Nude mice

## Abstract

Herein, we reported for the first time that RGD-conjugated silica-coated gold nanorods on the surface of multiwalled carbon nanotubes were successfully used for targeted photoacoustic imaging of *in vivo* gastric cancer cells. A simple strategy was used to attach covalently silica-coated gold nanorods (sGNRs) onto the surface of multiwalled carbon nanotubes (MWNTs) to fabricate a hybrid nanostructure. The cross-linked reaction occurred through the combination of carboxyl groups on the MWNTs and the amino group on the surface of sGNRs modified with a silane coupling agent. RGD peptides were conjugated with the sGNR/MWNT nanostructure; resultant RGD-conjugated sGNR/MWNT probes were investigated for their influences on viability of MGC803 and GES-1 cells. The nude mice models loaded with gastric cancer cells were prepared, the RGD-conjugated sGNR/MWNT probes were injected into gastric cancer-bearing nude mice models via the tail vein, and the nude mice were observed by an optoacoustic imaging system. Results showed that RGD-conjugated sGNR/MWNT probes showed good water solubility and low cellular toxicity, could target *in vivo* gastric cancer cells, and obtained strong photoacoustic imaging in the nude model. RGD-conjugated sGNR/MWNT probes will own great potential in applications such as targeted photoacoustic imaging and photothermal therapy in the near future.

## Background

Gastric cancer is the second most common cancer and the third leading cause of cancer-related death in China [1-3]. It remains very difficult to cure effectively, primarily because most patients present with advanced diseases [4]. Therefore, how to recognize and track or kill early gastric cancer cells is a great challenge for early diagnosis and therapy of patients with gastric cancer.

We have tried to establish an early gastric cancer pre-warning and diagnosis system since 2005 [5,6]. We hoped to find early gastric cancer cells *in vivo* by multimode targeted imaging and serum biomarker detection techniques [7-12]. Our previous studies showed that subcutaneous and *in situ* gastric cancer tissues with 5 mm in diameter could be recognized and treated by using multifunctional nanoprobes such as BRCAA1-conjugated fluorescent magnetic nanoparticles [13], her2 antibody-conjugated RNase-A-associated CdTe quantum dots [14], folic acid-conjugated upper conversion nanoparticles [15,16], RGD-conjugated gold nanorods [17], ce6-conjugated carbon dots [18], and ce6-conjugated Au nanoclusters (Au NCs) [19,20]. However, clinical translation of these prepared nanoprobes still poses a great challenge. Development of safe and highly effective nanoprobes for targeted imaging and simultaneous therapy of *in vivo* early gastric cancer cells has become our concern.

Carbon nanotubes (CNTs) have been intensively investigated due to their unique electrical, mechanical, optical, thermal, and chemical properties [21-26]. In the field of biomedical engineering, CNTs have shown promise as contrast agents for photoacoustic (PA) and photothermal imaging of tumors due to their strong near-infrared region (NIR) absorption and deep tissue penetration [27-29]. To date, single-walled carbon nanotubes (SWNTs) were fully investigated for photoacoustic imaging [30]. For example, for cell imaging, Avti et al. adopted photoacoustic microscopy to detect, map, and quantify the trace amount of SWNTs in different histological tissue specimens. The results showed that noise-equivalent detection sensitivity was as low as about 7 pg [31]. For *in vivo* PA imaging, Wu et al. adopted RGD-conjugated SWNTs as a PA contrast agent, and strong PA signals could be observed from the tumor in the SWNT-RGD-injected group [32]. With the aim of enhancing the sensitivity of the PA signal of SWNTs, Kim et al. developed one kind of gold nanoparticle-coated SWNT by depositing a thin layer of gold nanoparticles around the SWNTs for photoacoustic imaging *in vivo* and obtained enhanced NIR PA imaging contrast (approximately 10^2^-fold) [33-35]. However, to date, few reports are closely associated with the use of multiwalled carbon nanotubes (MWNTs) as a PA contrast agent. Therefore, it is very necessary to investigate the feasibility and effects of the use of MWNTs and gold nanorod-coated MWNTs as PA contrast agents. In addition, CNT-based *in vivo* applications have to consider their toxicity [36]. How to decrease or eliminate their cytotoxicity has become a great challenge. How to develop one kind of safe and effective NIR absorption enhancer MWNT has become our concern.

Gold nanorods (GNRs), because of their small size, strong light-enhanced absorption in the NIR, and plasmon resonance-enhanced properties, have become attractive noble nanomaterials for their potential in applications such as photothermal therapy [37], biosensing [38], PA imaging [39], and gene delivery [40] for cancer treatment. However, the toxicity derived from a large amount of the surfactant cetyltrimethylammonium bromide (CTAB) during GNR synthesis severely limits their biomedical applications. Therefore, removal of CTAB molecules on the surface of GNRs is an important step to avoid irreversible aggregation of GNRs and enhance their biocompatibility. In our previous work, we used a dendrimer to replace the CTAB on the surface of GNRs, markedly decreasing the toxicity of GNRs, and realized the targeted imaging and photothermal therapy [41]. We also used folic acid-conjugated silica-modified GNRs to realize X-ray/CT imaging-guided dual-mode radiation and photothermal therapy. Silica-modified GNRs can markedly enhance the biocompatibility of GNRs [42-44].

In recent years, molecular imaging has made great advancement. Especially, the system molecular imaging concept has emerged [45], which can exhibit the complexity, diversity, and *in vivo* biological behavior and the development and progress of disease in an organism qualitatively and quantitatively at a system level. Finally, system molecular imaging can enable the physicians to not only diagnose tumors accurately but also provide ‘on-the-spot’ treatment efficiently. In recent years, photoacoustic imaging, as an emerging imaging mode, has become a hotspot. We also synthesized gold nanoprisms and observed that gold nanoprisms could amplify the PA signal for *in vivo* bioimaging of gastrointestinal cancers [39]. However, how to obtain clear PA imaging of *in vivo* tumors and PA imaging-directed therapy to service clinical theranostics has become a great challenge.

**Figure 1:**
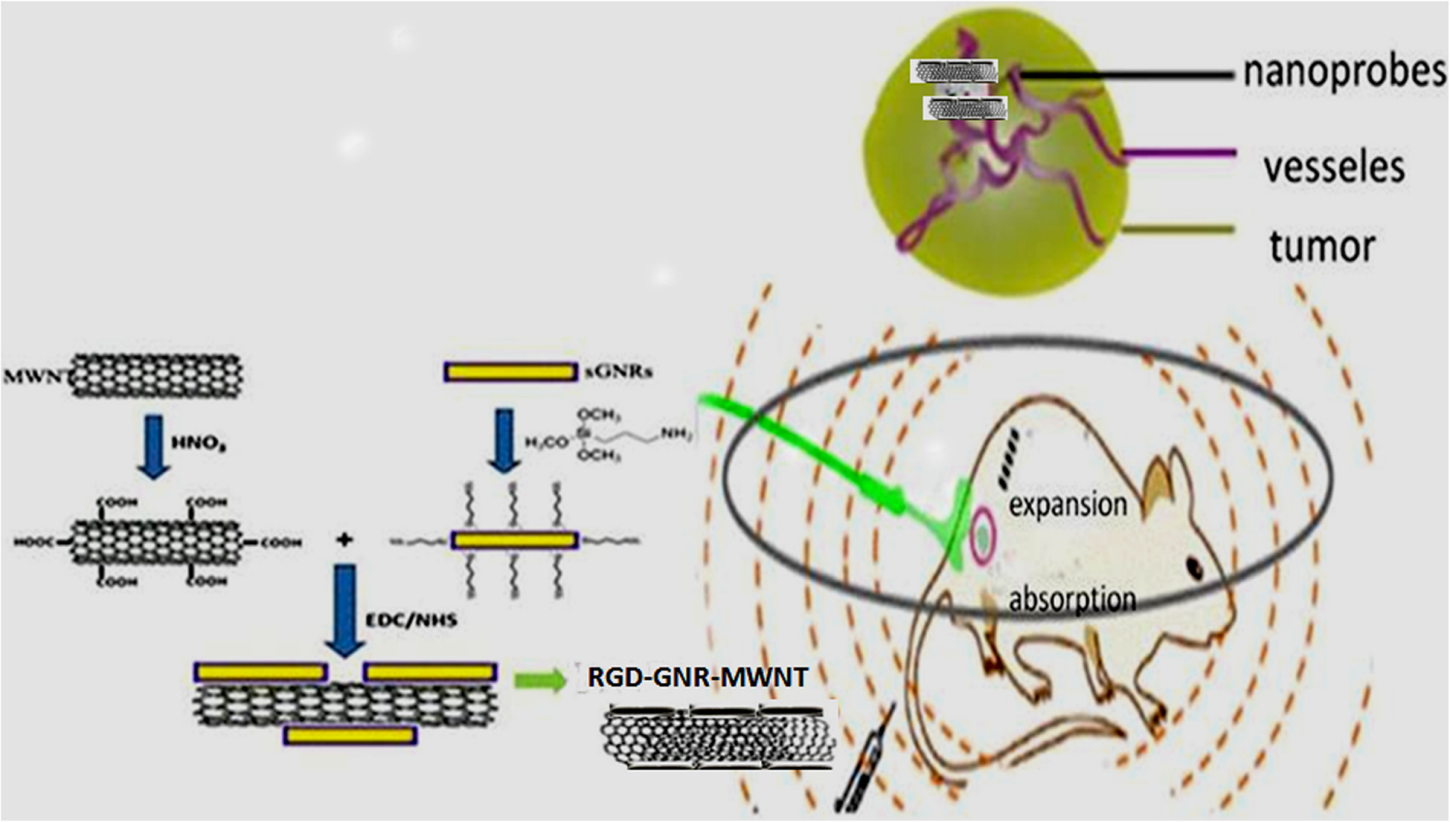
RGD-conjugated sGNR/MWNT hybrid for photoacoustic imaging.

Herein, we fully used the advantages of gold nanorods and multiwalled carbon nanotubes and developed a simple and effective strategy to prepare NIR absorption enhancer MWNTs through covalent interaction of carboxyl groups on the MWNTs with silica-coated gold nanorods (sGNRs). GNRs were prepared by the seed-mediated template-assisted protocol, coated by silica, and modified with the amino silane coupling agent with the aim of eliminating their cytotoxicity and improving their biocompatibility. Then, RGD peptides were conjugated with the sGNR/MWNT hybrid structure; resultant RGD-conjugated sGNR/MWNT (RGD-GNR-MWNT) nanoprobes were used for photoacoustic imaging of *in vivo* gastric cancer cells as shown in Figure 1. Our results showed that RGD-GNR-MWNT probes will own great potential in applications such as targeted PA imaging and photothermal therapy in the near future.

## Methods

All animal experiments (no. SYXK2007-0025) were approved by the Institutional Animal Care and Use Committee of Shanghai Jiao Tong University.

### Material source

Multiwalled carbon nanotubes (MWNTs) were purchased from the Shenzhen Nanoport Company (Shenzhen, China), and their diameters were around 20 ~ 30 nm. Chloroauric acid (HAuCl_4_ · 3H_2_O), cetyltrimethylammonium bromide (CTAB), sodium borohydride (NaBH_4_), tetraethylorthosilicate (TEOS), 3-aminopropyltrimethoxysilane (APTS), 1-ethyl-3-(3-dimethylaminopropyl)carbodiimide (EDC), *N*-hydroxysuccinimide (NHS), and ascorbic acid were obtained from Aldrich Company (Wyoming, IL, USA). Anhydrous ethanol and ammonium hydroxide were obtained from Sinopharm Co. (Beijing, China). RGD peptides were from Aldrich Company.

### Preparation of MWNT-COOH from MWNT

Crude MWNTs (0.523 g) were added to aqueous HNO_3_ (20.0 mL, 60%) (Figure 1). The mixture was placed in an ultrasonic bath (40 kHz) for 40 min and then stirred for 48 h while being boiled under reflux. The mixture was then vacuum-filtered through a 0.22-mm Millipore polycarbonate membrane (Millipore Co., Billerica, MA, USA) and subsequently washed with distilled water until the pH of the filtrate was *ca.* 7. The filtered solid was dried under vacuum for 24 h at 70°C, yielding MWNT-COOH (0.524 g) [46,47].

### Synthesis of silica-modified gold nanorods

In a typical experiment, GNRs were synthesized according to the seed-mediated template-assisted protocol [11,48]. Twenty milliliters of the GNR solution was centrifuged at 9,600 rpm for 15 min. The supernatant, containing mostly CTAB molecules, was removed and the solid (containing rods) was redispersed in 20 mL anhydrous ethanol adjusted to pH 10 with ammonia. After the system was sonicated for 30 min, TEOS of 4 mL (10 mM) was added to the above system and the entire system was stirred for 20 h. Next, 10 mL APTS was added to form a mixed solution and allowed to react at 80°C for 3 h. The resultant product was treated by high-speed centrifugal separation and washed with deionized water for several times, and then dried at 60°C for 3 h in a vacuum oven to obtain the sGNRs.

### Fabrication of sGNR/MWNT nanohybrid

Covalent attachment of sGNRs to the MWNTs was performed using a modification of the standard EDC/NHS reaction [49,50]. Carboxyl groups on the surface of MWNTs (5 mg) were activated by an EDC/NHS solution for 30 min. Following activation, 1 mg of sGNRs were added to form a mixed solution and allowed to react at room temperature for 6 h, and then RGD peptides were added into the mixed solution and continued to react at room temperature for 6 h. The resultant products were treated by high-speed centrifugal separation and washed with deionized water for three times, and then kept at 4°C for use.

### Characterization of sGNR/MWNT nanohybrid

A JEOL JEM-2010 transmission electron microscope and a JEOL JEM-2100 F high-resolution transmission electron microscope (JEOL Ltd., Akishima, Tokyo, Japan) were used to confirm particle size and observe the interface and the binding site of sGNRs and MWNTs. UV-vis spectra were measured at 20°C with a Shimadzu UV-2450 UV-visible spectrophotometer (Shimadzu Corporation, Kyoto, Japan) equipped with a 10-mm quartz cell, where the light path length was 1 cm. The 200- to 1,000-nm wavelength region was scanned, since it includes the absorbance of the GNRs. The Fourier transform infrared (FTIR) spectra were recorded on a PerkinElmer Paragon-1000 FTIR spectrometer (PerkinElmer, Waltham, MA, USA). Zeta potential was measured with a Nicomp 380ZLS Zeta Potential/Particle Sizer (Nicomp, Santa Barbara, CA, USA).

### Effects of RGD-GNR-MWNT nanoprobes on cell viability

Effects of RGD-GNR-MWNT nanoprobes on viability of MGC803 and GES-1 cells were analyzed using Cell Counting Kit-8 (CCK8) assay [23]. MGC803 and GES-1 cells were cultured in a 96-well microplate at the concentration of 5,000 cells per well and incubated in a humidified 5% CO_2_ balanced air incubator at 37°C for 24 h. Except for control wells, the remaining wells were added into the medium with RGD-GNR-MWNT nanoprobes. Final concentrations were, respectively, 5, 10, 40, and 80 μg/mL, and then those cells were continuously cultured for 24 days. Then, the ODs were measured using the Thermo Multiskan MK3 ELISA plate reader (Thermo Fisher Scientific, Waltham, MA, USA) according to the protocol of the CCK8 assay kit, and the survival rate of cells was calculated. The survival rate of cells can be calculated using the following equation:

$$\begin{array}{l} \mathrm{Cell}\;\mathrm{viability}\;\left(\%\right)\;=\mathrm{Optical}\;\mathrm{density}\;\left(\mathrm{OD}\right)\;\mathrm{of}\;\mathrm{the}\;\mathrm{treated}\;\mathrm{cells}\\ \;\;/\mathrm{OD}\;\mathrm{of}\;\mathrm{the}\;\mathrm{non}‒\mathrm{treated}\;\mathrm{cells}\;\times \;100 \end{array}$$

### Nanoprobes for *in vitro* targeted imaging of gastric cancer cells

Gastric cancer cell line MGC803 used as target cells and human gastric mucous GES-1 used as control cells were cultured and collected [12-15], and then were treated with 50 μg/mL of prepared nanoprobes and cultured in a humidified 5% CO_2_ balanced air incubator at 37°C for 4 h. Meanwhile, the MGC803 and GES-1 cells treated with the prepared probes were used as the control group. Afterward, the cells were rinsed with phosphate buffered saline (PBS) three times and then fixed with 2.5% glutaraldehyde solution for 30 min. For nuclear counterstaining, MGC803 cells were incubated with 1 mM Hoechst 33258 in PBS for 5 min. The cells were observed and imaged using a fluorescence microscope (Nikon TS100-F, Nikon Co., Tokyo, Japan).

### Preparation of gastric cancer-bearing nude mice model

Pathogen-free athymic nude (nu/nu) BALB/c mice were housed in an accredited vivarium, maintained at 22°C ± 0.5°C with a 12-h light/dark cycle and were allowed to access food and water. Male athymic nude mice (4 to 6 weeks old) were used to establish subcutaneous gastric cancer models; 2 × 10^6^ MGC803 cells suspended in 100 μL of pure DMEM were subcutaneously injected into the right anterior flank area of each mouse. Four weeks later, tumors were observed to grow to approximately 5 mm in diameter.

### RGD-conjugated sGNR/MWNT nanoprobes for photoacoustic imaging

Photoacoustic imaging of the study *in vitro* and *in vivo* was accomplished by a PA system (Endra Nexus 128, Endra Life Sciences, Ann Arbor, MI, USA). The excitation laser (Opotek, Carlsbad, CA, USA) is irradiated from the bottom of a hemispherical bowl, whose wavelength is tunable from 680 to 950 nm. PA characteristics of prepared nanoprobes *in vitro* were firstly investigated before *in vivo* imaging. PA intensity corresponding to different concentrations and wavelengths were studied by setting the probe in the tube. Subsequently, gastric cancer-bearing nude mice were treated with 500 μg of prepared nanoprobes. Animal orientation and tumor position should be kept constant in the bowl during experiments to make sure that each scan was in the same position in favor of comparison and imaging alignment. Filling the slot with distilled water provided acoustic coupling with the animal. Then, pre-injection scans and post-injection scans were both acquired when the tumor site was irradiated by the laser. The PA signals, which were received by the ultrasonic transducers, were spirally distributed on the surface of the bowl and then directed to a computer. Reconstruction of the 2D and 3D PA image was performed using Osirix imaging software (OsiriX Foundation, Geneva, Switzerland).

## Results and discussion

### Preparation and characterization of sGNR/MWNT hybrid

**Figure 2:**
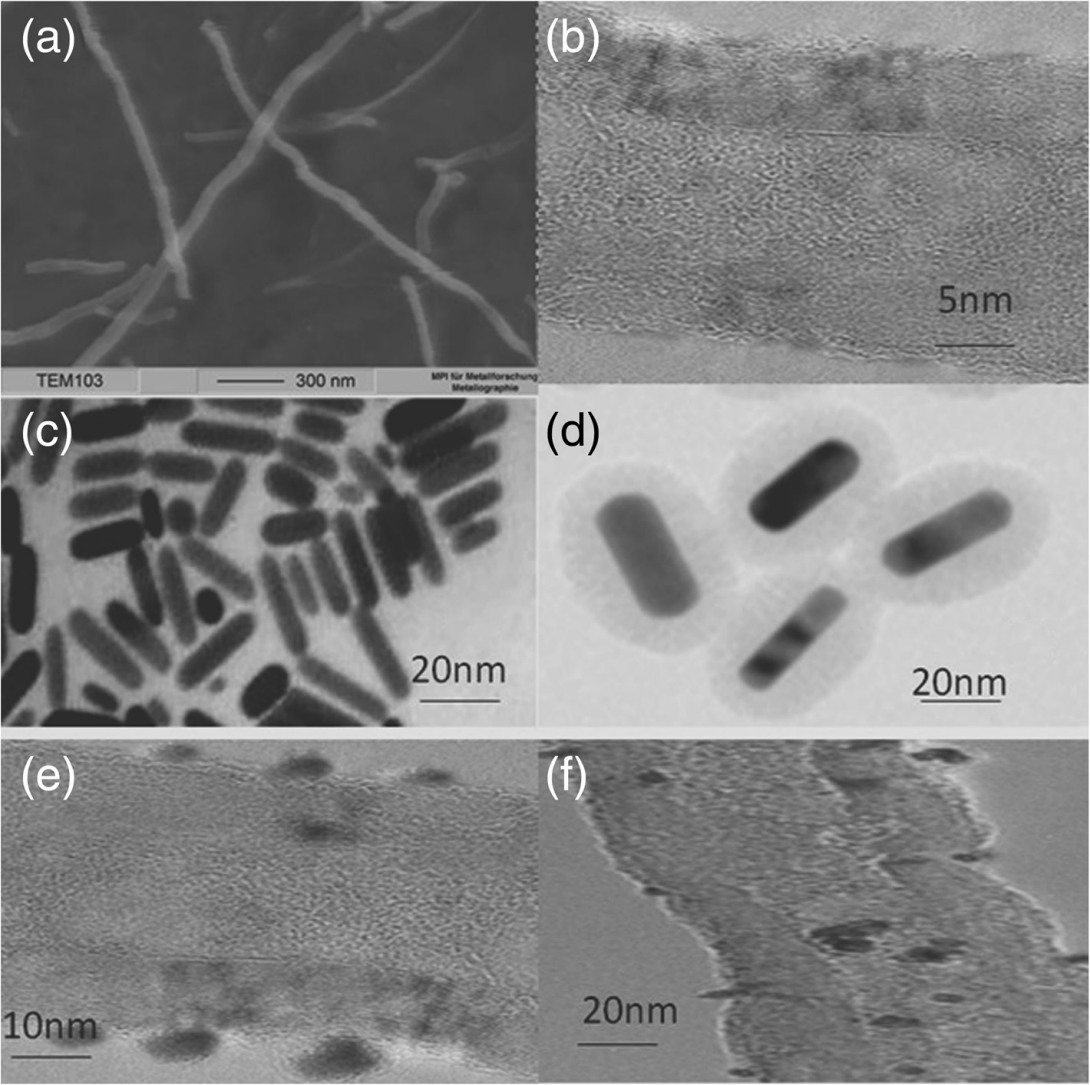
**TEM and HR-TEM images. (a, b)** MWNTs, **(c, d)** sGNRs, and **(e, f)** MWNTs/sGNRs.

Figure 2 showed typical transmission electron microscopy (TEM) images and high-resolution TEM (HR-TEM) images of (a, b) MWNTs, (c, d) sGNRs, and (e, f) MWNTs/sGNRs. As shown in Figure 2a, MWNTs are very pure and did not contain amorphous carbon particles, metal catalysts, etc. The average diameter of MWNTs was around 20 nm. Figure 2b showed the highly crystalline nature of MWNTs (see Additional file 1). Figure 2c showed the morphology and the size distribution of silica-coated GNRs; the sGNRs were approximately spherical with a size of about 80 nm. The sGNRs exhibited monodispersed, well-defined core-shell structures. The GNR core, with 50 nm in length and 20 nm in width, was prepared by seed-mediated template-assisted method. The silica shell has a thickness of 10 to 20 nm. Figure 2d is the HR-TEM image of an individual sGNR, showing that the silica shell has a well-ordered mesopore structure. Figure 2e,f showed that the sGNRs combined on the surface of MWNTs mainly along their sidewalls, highly suggesting that sGNRs successfully attached to MWNTs. The well-distributed sGNRs deposited onto the surface of MWNTs showed that the CNT pre-treatment was effective, which resulted in many active sites on the MWNTs. Figure 2f showed that the structure and the crystallinity of MWNTs and sGNRs did not change after the cross-link. Almost 90% of sGNRs were successfully cross-linked with MWNTs; the average size of RGD-sGNRs/MWNTs was almost 300 nm in length and 50 nm in width.

### Binding sites of sGNRs and MWNTs

**Figure 3:**
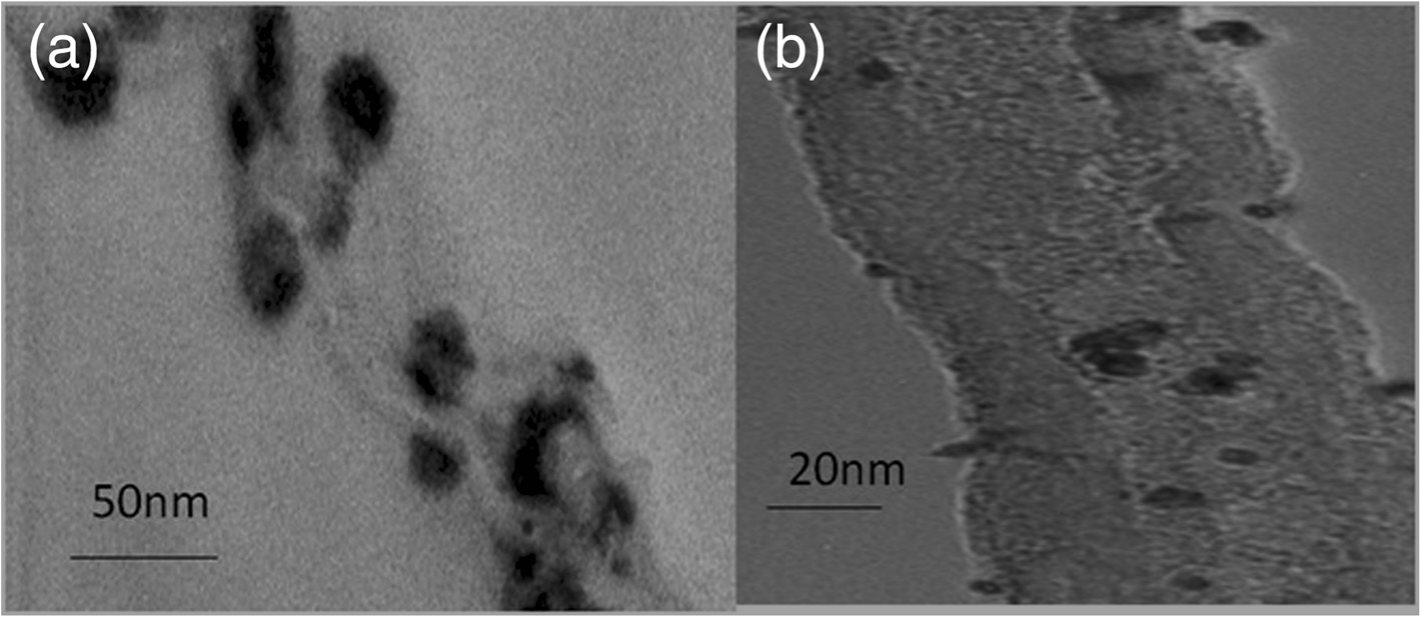
**TEM images of the different binding sites of sGNRs and MWNTs. (a)** sGNRs attached on the surface of WNT along the sidewalls. **(b)** sGNRs attached on the end of WNT.

Figure 3 showed TEM images of the different binding sites of sGNRs and MWNTs. According to the TEM observations, the sGNRs decorated the surface of MWNTs mainly along their sidewalls (Figure 3a) and partly connected to the WNT ends (Figure 3b), which may be attributed to the fact that the amount of amino groups on the long axis of GNRs is more than the amount on the short axis of GNRs.

### UV-vis spectra of gold nanorods

**Figure 4:**
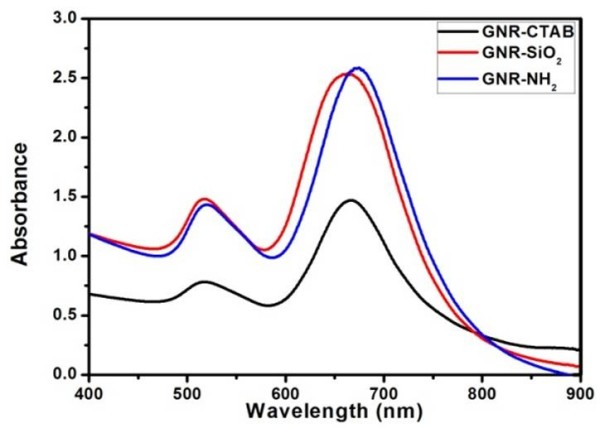
**UV-vis spectra of GNR-CTAB, GNR-SiO**_**2**_**, and GNR-NH**_**2**_**.**

**Figure 5:**
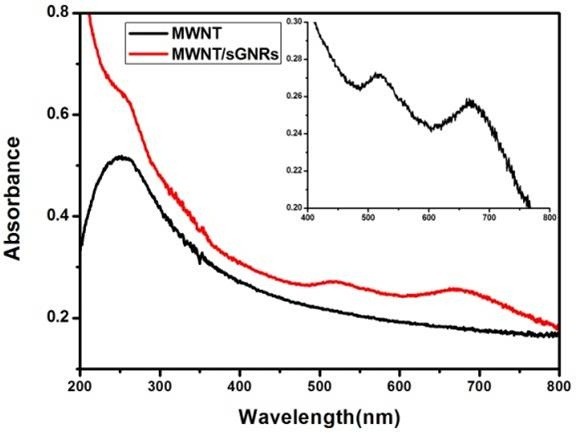
**UV-vis spectra of MWNTs and MWNTs/sGNRs.** The inset shows the magnification in the region of 400 ~ 800 nm.

Figure 4 showed the UV-vis absorbance spectra of GNR-CTAB, GNR-SiO_2_, and sGNRs in the wavelength range of 400 ~ 900 nm. The spectrum of GNR-CTAB showed that GNR-CTAB had two absorption bands: a weak short-wavelength band around 515 nm and a strong long-wavelength band around 715 nm. Moreover, we observed that the plasmon peaks of GNR-SiO_2_ exhibited no significant changes in peak width or position, so the silica modification could improve only the biocompatibility of GNRs and did not change the two absorption bands of GNRs. After being modified with the second amino silane coupling agent, the special absorption peaks of sGNRs exhibited a little redshift (approximately 6 nm), which may be attributed to the fact that the coated silica layer became thick and the size of sGNRs became big.Figure 5 showed the UV-vis absorbance spectra of MWNTs and sGNRs/MWNTs. MWNTs exhibited a relative low absorption peak at NIR, and after MWNTs covalently bound with sGNRs, the sGNRs/MWNTs exhibited marked NIR absorption enhancement. The inset showed the magnification absorbance spectra of sGNRs/MWNTs in the region of 400 ~ 800 nm, where there existed two special absorption peaks matched with sGNRs.

### FTIR spectroscopy of RGD-conjugated GNR/MWNT nanoprobes

**Figure 6:**
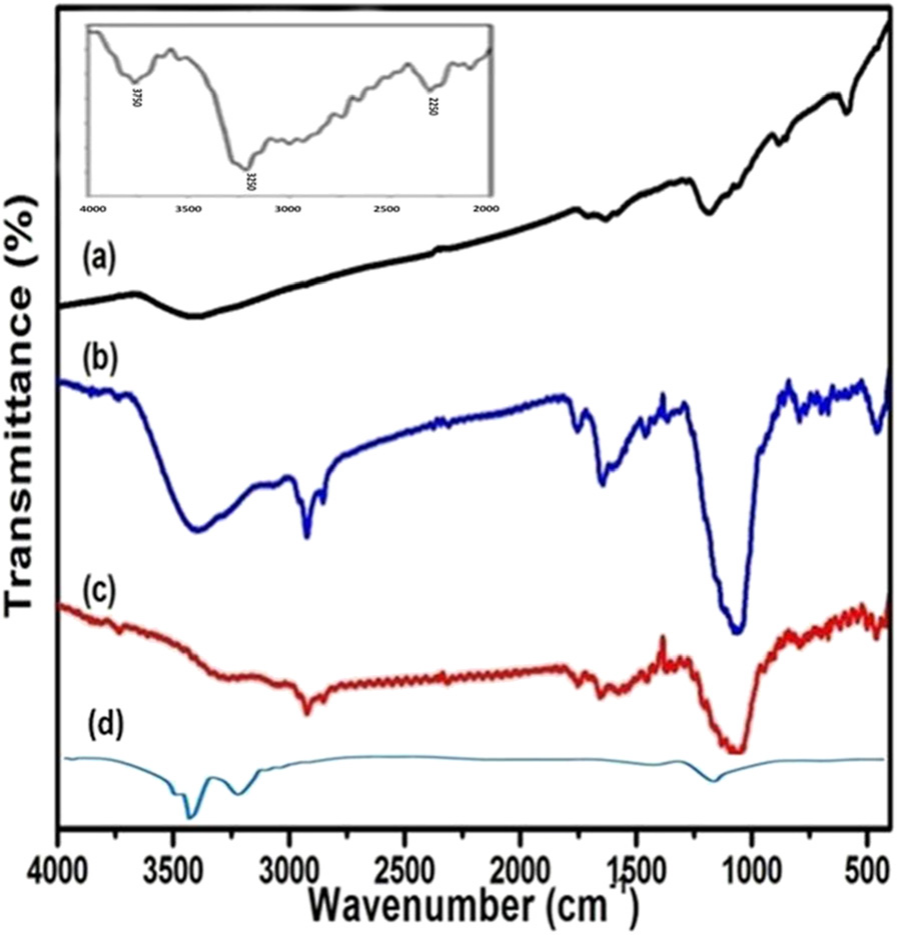
FTIR spectra of (a) MWNTs, (b) sGNRs, (c) sGNRs/MWNTs, and (d) RGD-GNR-MWNT.

Figure 6 showed the typical FTIR spectra of (a) MWNTs, (b) sGNRs, (c) sGNRs/MWNTs, and (d) RGD-MWNT/sGNR. The presence of sGNRs can be seen by a strong absorption band at around 1,060 cm^-1^. In addition, Figure 6 (a) and (b) showed the absorption bands near 3,400 and 1,630 cm^-1^, referring to the vibration of the remaining H_2_O in the samples. The fact was proven by comparison of FTIR spectra of the MWNTs and sGNR/MWNT nanohybrids shown in Figure 6 (a) and (c). The difference between the IR spectrum of MWNTs and that of MWNTs/sGNRs is obvious. The Si-O band at 1,061 cm^-1^ indicated the silica in (c), but it was not found in (a). Covalent attachment of sGNRs to MWNTs was verified by pronounced amide I and III vibrational stretches (1,641 and 1,462 cm^-1^, respectively, Figure 6 (inset)). These changes in FTIR absorption spectroscopy can be explained by the covalent interaction between sGNRs and MWNTs. Figure 6 (d) showed that the FTIR of RGD-conjugated MWNTs/sGNRs, peaks observed at 3,200 and 3,450 cm^-1^, indicated that RGD peptides had been successfully grafted onto the surface of MWNTs/sGNRs.

### Effects of RGD-GNR-MWNT on cell viability

**Figure 7:**
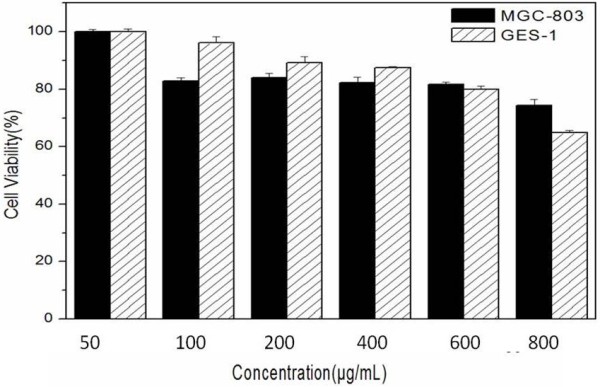
Effects of RGD-GNR-MWNT nanoprobes on cell viability.

Regarding the effects of RGD-GNR-MWNT on MGC803 and GES-1 cells, as shown in Figure 7, RGD-GNR-MWNT affected the growth of MGC803 and GES-1 cells in dose-dependent means. RGD-GNR-MWNT probes with a concentration of 50 μg/mL in the medium exhibited no cellular toxicity; the cell survival rate increased with the increase of culture days. When the dose of RGD-GNR-MWNT probes in the medium reached or overrun 800 μg/mL, RGD-GNR-MWNT probes exhibited low cytotoxicity to MGC803 cells, the cell growth became slow, and there existed a statistical difference between the test group and control group (*P* < 0.05). Thus, we consider that RGD-GNR-MWNT nanoprobes exhibited good biocompatibility to MGC803 and GES-1 cells within the dose of 800 μg/mL in the medium.

### RGD-GNR-MWNT nanoprobes for *in vitro* cell targeted imaging

**Figure 8:**
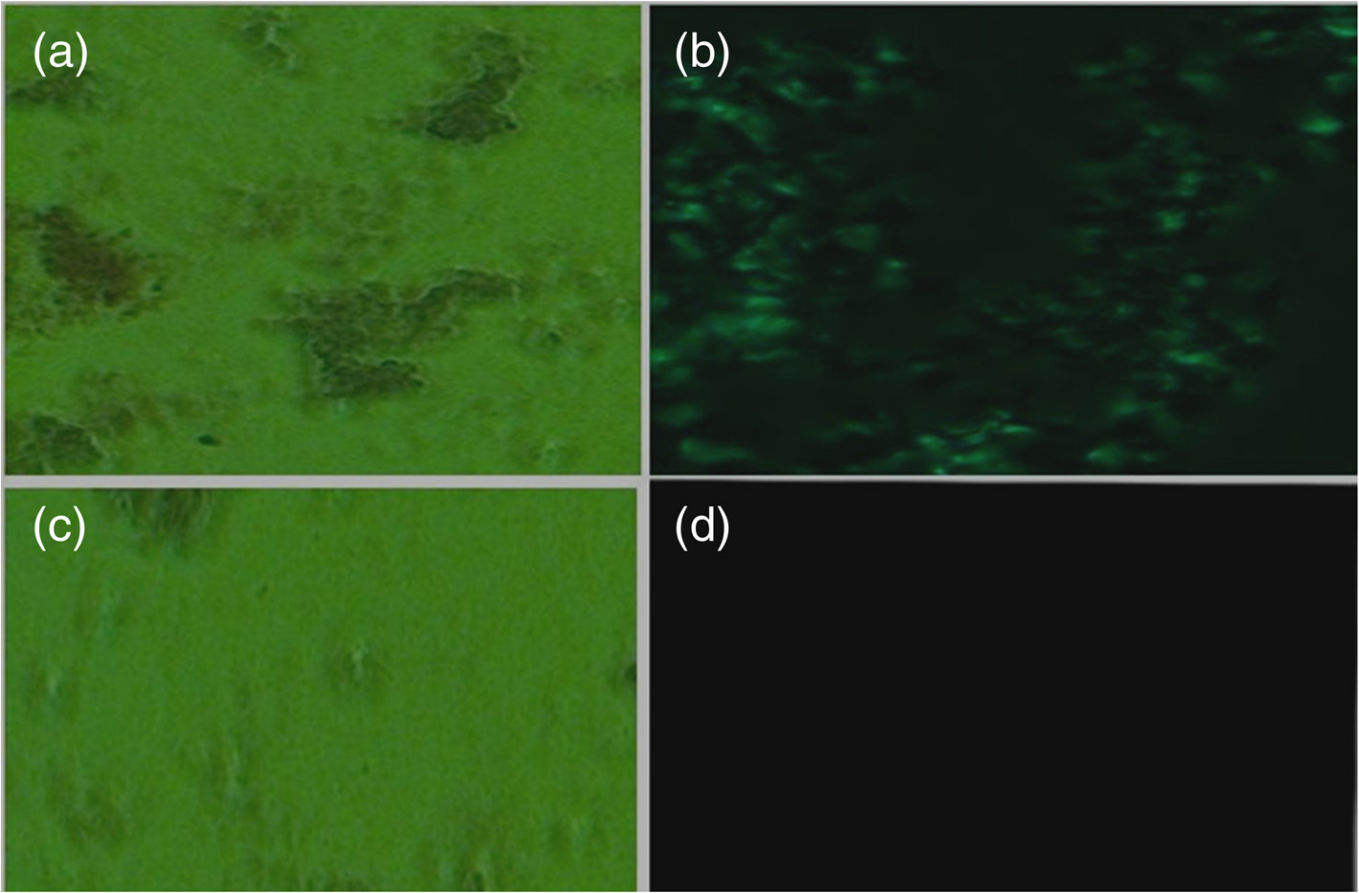
**RGD-GNR-MWNT nanoprobes for *in vitro *cell targeted imaging. (a)** MGC803 cell imaged under bright-field microscopy. **(b)** MGC803 cell imaged under dark-field microscopy. **(c)** GES-1 cell imaged under bright-field microscopy. **(d)** GES-1 cell imaged under dark-field microscopy.

As shown in Figure 8, gastric cancer cell line MGC803 cells were used as target cells and human gastric mucous GES-1 cells were used as control. Prepared RGD-GNR-MWNT nanoprobes could target MGC803 cells. Under dark-field microscopy, MGC803 cells exhibited a golden color, whereas GES-1 cells exhibited no golden color, which indicated that the prepared RGD-GNR-MWNT nanoprobes could target MGC803 cells; because RGD only displayed overexpression on the surface of MGC803 cells, there was no expression on the surface of GES-1 cells [51]. Therefore, the prepared RGD-GNR-MWNT nanoprobes could target gastric cancer MGC803 cells.

### RGD-GNR-MWNT nanoprobes for *in vivo* photoacoustic imaging

**Figure 9:**
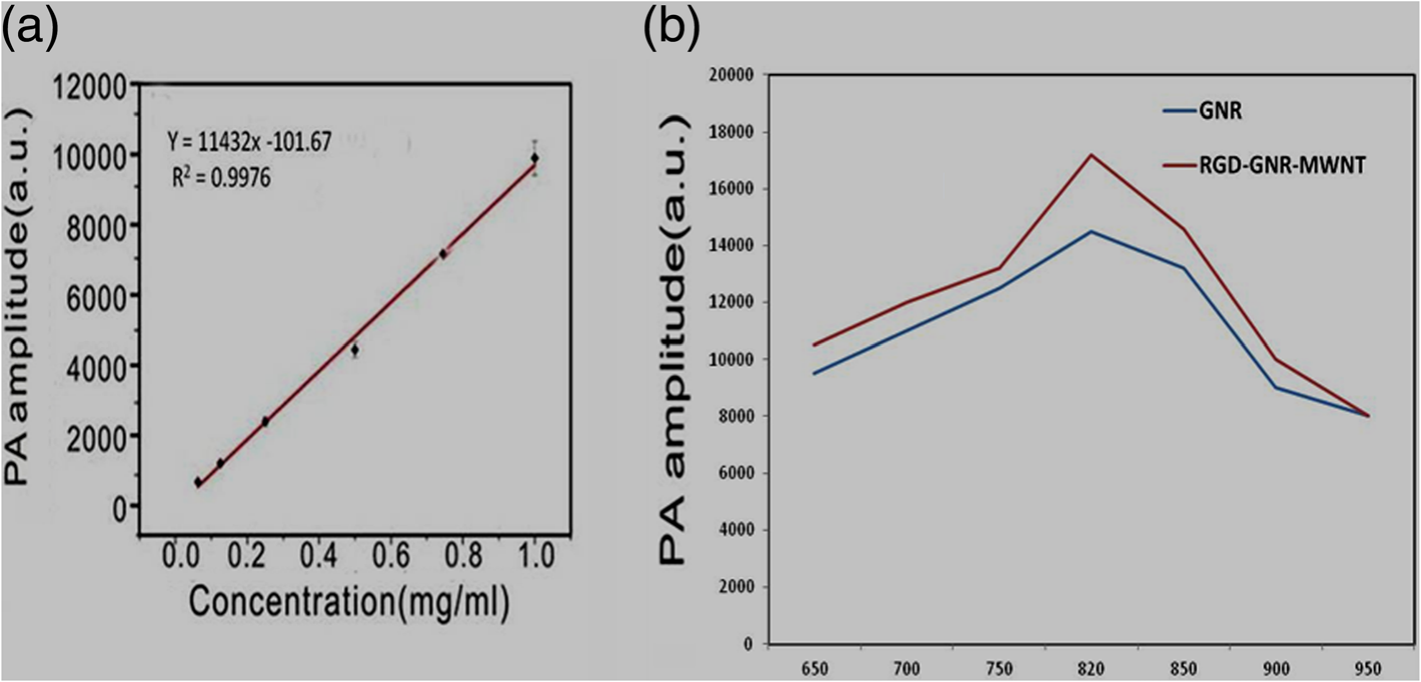
**Relationship curves. (a)** Relationship curve between nanoprobe concentration and PA signal intensity. **(b)** Gold nanorod-enhanced MWNT PA signal amplitude curve at different wavelengths (black, sGNRs; red, RGD-sGNR/MWNTs).

Multispectral optoacoustic tomography (MSOT) is a rapidly emerging, noninvasive, and high-resolution photoacoustic imaging system which can achieve an isotropic and homogeneous spatial resolution of 200 μm. A near-infrared pulse laser serving as the excitation source receives PA signals for three-dimensional (3D) image reconstruction [30,52]. RGD-conjugated sGNR/MWNT nanoprobes were applied to photoacoustic imaging to detect gastric cancer cells in *in vivo* subcutaneous gastric cancer xenograft model. As shown in Figure 9a, as the concentration of prepared nanoprobes increased, PA signal amplitudes also increased correspondingly. As shown in Figure 9b, compared with GNRs, RGD-sGNR/MWNT composites could markedly enhance the MWNT PA signals at about 20%, which highly suggests that sGNRs could enhance the PA imaging signal of MWNTs.

**Figure 10:**
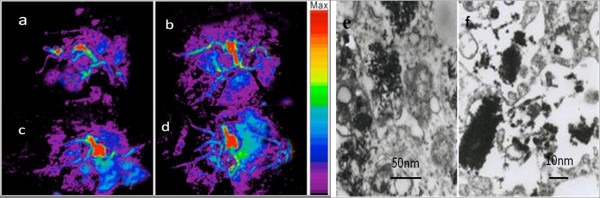
**The prepared nanoprobes for photoacoustic imaging of *in vivo *gastric cancer cells.** Photoacoustic images at **(a)** 1 h, **(b)** 3 h, **(c)** 6 h, and **(d)** 12 h post-injection. **(e,f)** TEM pictures of prepared nanoprobes located inside MGC803 cells.

As shown in Figure 10a,b,c,d, as the post-injection time increased, the prepared nanoprobes could target actively vessels of *in vivo* gastric cancer tissues and accumulated more and more in the site of gastric cancer tissues. The photoacoustic signals of tumor vessels became stronger, and photoacoustic amplitudes reach the maximum at the 850-nm wavelength. Figure 10e,f showed prepared nanoprobes located inside the MGC803 cells. Our results fully demonstrate that RGD-conjugated sGNRs/MWNTs may be a good contrast agent for photoacoustic imaging of *in vivo* gastric cancer cells, and gold nanorods can enhance the PA signal of MWNTs. Golden single-walled carbon nanotubes have been used for PA imaging of *in vivo* tumors [30,33]. Compared with available data, gold nanorod-modified multiwalled carbon nanotubes exhibited enhanced PA signals. Gold nanorods may have minor advantages over thin gold nanolayer for enhanced PA signals of carbon nanotubes.

## Conclusions

In summary, we for the first time designed and prepared RGD-conjugated MWNT/sGNR nanoprobes, demonstrated that GNRs can enhance the PA signal of multiwalled carbon nanotubes and that RGD-conjugated MWNT/sGNR nanoprobes have good biocompatibility and can be used to target *in vivo* tumor vessels, and realized enhanced MWNTs' PA imaging of tumor vessels. Our results also confirm that MWNTs may be good PA imaging contrast agents. Although prepared RGD-conjugated MWNT/sGNR nanoprobes' distribution and metabolism are not clarified well, the novel hybrid nanostructure should open up new possibilities in nanomedicine as a multimodal photoacoustic and photothermal contrast agent, and will have great potential applications in advanced sensing, photoacoustic imaging, and photothermal therapy in the near future.

## Competing interests

The authors declare that they have no competing interests.

## Authors' contributions

WC carried out nanoprobe preparation and animal experiments. BC finished the characterization of CNTs and GNRs. LC finished the surface modification of MWNTs and GNRs. DM and FH finished the RGD conjugation with the surface of GNRs. WK and CD finished the result analysis. FH and WC finished the draft. LQ and CD finished the experiment design and manuscript revision. All authors of this paper have read and approved the final manuscript.

## Supplementary Material

Additional file 1 **Supplementary figures.** A document showing the Raman spectra of MWNTs (black, untreated; red, treated with HNO_3_) (**Figure S1**) and TEM image of RGd-sGNR/MWNT located inside the cytoplasm (**Figure S2**). Click here for file
