# Supplementary material for: RGD-conjugated silica-coated gold nanorods on the surface of carbon nanotubes for targeted photoacoustic imaging of gastric cancer
Source: Nanoscale Res Lett. 2014 May 27;9(1):264. doi: 10.1186/1556-276X-9-264 (PMC4053550; doi:10.1186/1556-276X-9-264)

**Additional file 1**

Figure S1 Raman Spectra of MWNTs ( black: untreated, red: treated with HNO3.)





Figure S2 TEM image of RGd-sGNRs/MWNT located inside cytoplasm


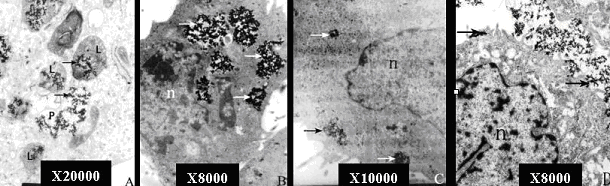

Supplement: Additional file 1 — Supplementary figures. A document showing the Raman spectra of MWNTs (black, untreated; red, treated with HNO3) (Figure S1) and TEM image of RGd-sGNR/MWNT located inside the cytoplasm (Figure S2). [file 1556-276X-9-264-S1.docx]
